# Beyond bricks and mortar: a rural network approach to preclinical medical education

**DOI:** 10.1186/1472-6920-14-166

**Published:** 2014-08-09

**Authors:** Douglas L Myhre, Paul Adamiak, Nathan Turley, Ron Spice, Wayne Woloschuk

**Affiliations:** 1 Department of Family Medicine, University of Calgary, Faculty of Medicine, Calgary, Alberta, Canada; 2 University of Calgary Faculty of Medicine in Calgary, HMRB G17, 3330 Hospital Drive NW, Calgary, AB T2N 4 N1, Canada; 3 Department of Family Medicine, University of Calgary, Calgary, Alberta, Canada; 4 Division of Palliative Medicine, University of Calgary Faculty of Medicine, Calgary, Alberta, Canada; 5 Faculty of Medicine at the University of Calgary, Calgary, Alberta, Canada

**Keywords:** Pre-clinical medical education, Distributed medical education, Rural medical education, Academic equivalence, Social learning

## Abstract

**Background:**

Countries with expansive rural regions often experience an unequal distribution of physicians between rural and urban communities. A growing body of evidence suggests that the exposure to positive rural learning experiences has an influence on a physician’s choice of practice location. Capitalizing on this observation, many medical schools have developed approaches that integrate rural exposure into their curricula during clerkship. It is postulated that a preclinical rural exposure may also be effective. However, to proceed further in development, accreditation requirements must be considered. In this investigation, academic equivalence between a preclinical rural community based teaching method and the established education model was assessed.

**Method:**

Two separate preclinical courses from the University of Calgary’s three year Undergraduate Medical program were taught at two different rural sites in 2010 (11 students) and 2012 (12 students). The same academic content was delivered in the pilot sites as in the main teaching centre. To ensure consistency of teaching skills, faculty development was provided at each pilot site. Academic equivalence between the rural based learners and a matched cohort at the main University of Calgary site was determined using course examination scores, and the quality of the experience was evaluated through learner feedback.

**Results:**

In both pilot courses there was no significant difference between examination scores of the rural distributed learners and the learners at the main University of Calgary site (*p* > 0.05). Feedback from the participating students demonstrated that the preceptors were very positively rated and, relative to the main site, the small group learning environment appeared to provide strengthened social support.

**Conclusion:**

These results suggest that community distributed education in pre-clerkship may offer academically equivalent training to existing traditional medical school curricula while also providing learners with positive rural social learning environments. The approach described may offer the potential to increase exposure to rural practice without the cost of constructing additional physical learning sites.

## Background

Canada, with its expansive landmass and disproportionately small population, experiences a shortage of rural physicians. In the province of Alberta alone, 14% of all physicians practice in rural communities [1], despite ~20% of the population representing these communities [2]. In an effort to respond to the social contract with their communities, many medical schools have sought to address this disparity by increasing learners’ exposure to rural practice and generalist disciplines [3,4]. At the University of Calgary (UC) and other medical centres, the Longitudinal Integrated Clerkship (LIC), rural clerkships, and rural based postgraduate training programs have been seen as promising initiatives in promoting rural practice [5-8].

Various models of distributed medical education have been described in the literature; each seeks to address geographic disparities in physician resources. Some programs have constructed medical schools or designated buildings as enduring sites for medical training. The Washington, Wyoming, Alaska, Montana and Idaho Regional Medical Education Program (WWAMI) educates medical students through an “institutional matrix” of satellite medical schools and community exposure in clerkship [9]. Similarly, the Indiana University School of Medicine (IUSM) has operated preclinical regional schools since the late 1960s [10] and the University of British Columbia (UBC) also distributes its undergraduate class across four campuses [11].

A contrasting strategy is teaching and housing medical students within community. The Northern Ontario School of Medicine (NOSM) [12], provides students with early exposure to rural practice and generalist disciplines through community-based medical education. The UC model described here was influenced by the NOSM medical education model, but is distinct as it focuses on preclinical medical students, uses local generalists to teach courses, and, unlike the satellite models, employs facilities not specifically created for medical education. The UC program was designed to function as a network of independent sites rather than a building based program, and is uniquely distinct from the satellite medical school models, as well as from the NOSM model, as it seeks to integrate community based medical education into an existing traditional medical school.

The purpose of this investigation was to gather preliminary evidence of the impact of the new teaching model on students, both academically and socially. Whenever new educational delivery models are considered, accreditation standards require that academic performance and learner well-being are proven equivalent to existing models. In this case, the delivery of academic content at a rural community in the absence of a previously designated stand-alone medical education facility was examined. The pilot projects sought to determine whether offering a portion of the preclinical curriculum in rural/regional sites, taught by generalists, would allow students to achieve academic equivalence and whether learners self-reported social impacts specific to the smaller teaching locations. Two distinct courses were considered and delivered, as it was felt necessary to use more than one discipline or curriculum to verify early indications.

The UC is an intensive, three-year medical school with a clinical presentation based curriculum. Two pilot projects (October 2010 and January 2012) in two different disciplines, Course 5 (Neurology, Pain and Elderly) and Course 7 (Mind and Family), were delivered to determine whether an existing traditional *preclinical* curriculum model could support a distributed or networked medical education system in parallel to its main site in Calgary.

A steering committee consisting of academic student representatives, the Preclerkship Assistant Dean, the Associate Dean for Distributed Learning, and the Undergraduate Medical Education Evaluator was formed to develop the pilot program. The initial development of the UC Preclinical Networked Medical Education (PNME) pilot focused on curricular review for networked academic delivery. Courses that were not in transition, as defined by having stable leadership and curriculum, and that already incorporated some use of electronic delivery were approached regarding the project. Once the course Chairs had agreed to the project, the course committees conducted curricular review with the understanding that generalist preceptors would deliver the same clinical core and small group components as in the main site, and that didactic lectures would be accessible to all learners via podcast. Faculty development was required at all sites prior to learner arrival. The assessment tools for each course remained unchanged between the traditional setting and the pilot sites. The selected course Chairs then joined the membership of the Organizing Committee.

Teaching communities were considered once courses were approved. Experienced teaching sites were reviewed for participation based on three selection requirements. The community hospital and clinic required either adequate patient activity for the courses selected or had the potential for patient recruitment. Sites also required generalist community preceptors willing to provide clinical training and mentorship to students. Lastly, preference was given to sites that offered consistent Internet access and student study space.

As the pilot sites were being selected, the PNME pilot was advertised to UC medical students to attract potential participants. The students who expressed interest in the pilot met with committee representatives to discuss and learn about the pilots. These regular information meetings were held to probe students’ ideas for the PNME and to address potential applicant concerns such as housing or IT connectivity. Because there were more applicants than there were placements for the PNME, those students participating in the trial were randomly selected from a pool of volunteer applicants.

The first pilot was conducted in October 2010 at two sites, site A and site B. Site A is a regional centre with a population of ~85000 and site B is a rural centre with a population of ~8500. This pilot involved teaching a half of Course 5 to a group of second-year medical students using the resources existing at both sites. Modules requiring access to anatomy labs and simulations were taught at the main site. The goal of employing local resources was to deliver the academic content at site without a previously designated stand-alone medical education facility. The second pilot was deployed for Course 7 only at site A in January 2012. Students were taught for three weeks at the distributed site and for one week at the main site. A different course was deliberately chosen for the second pilot to verify that the observations were a result of the academic delivery method and not the particular course content.

## Methods

Students in the pilot courses were placed at one of the rural/regional sites for a period of three weeks. Each course curriculum was delivered consistently and concurrently at all sites through videoconferencing of lectures from Calgary, with backup podcasts for review. Both urban and rural groups attended small group discussions and prescribed clinical sessions. Local generalist preceptors facilitated small group sessions for the study group. This differs from the urban course format where specialists facilitated the small group sessions.

Quantitative methods were used to assess the academic equivalence of the distributed learning experience. Four control students were matched to each pilot student based on the cumulative percentage grade point average on the courses prior to Course 5 and Course 7, respectively for the different pilot implementations. Academic equivalency between rural students and the matched cohort of classmates in Calgary was assessed using summative examination scores for Course 5 and Course 7. Data were analyzed in STATA v12.0 using student’s independent *t* test, and an alpha of 0.05, to compare course examination scores between the students at the main site in Calgary and the rural distributed sites. Mean change scores within student cohorts (distributed and at the main site) before and after the pilot were calculated to investigate potential effects from motivation factors amongst the students being studied.

After completing the course, students’ subjective experiences were evaluated through semi structured interviews conducted by an independent external interviewer. Two investigators reviewed 11 interview transcripts for the first pilot, and 12 for the second. Each investigator independently identified emergent themes, which were then collectively evaluated. Consistently important themes were developed by consensus amongst investigators.

The evaluation of each distributed learning pilot was granted IRB approval by the University of Calgary Conjoint Health Research Ethics Board. Approval was received on June 11 2010 and January 12 2013, for the investigation of Course 5 and Course 7 courses respectively.

## Results

Investigation of the summative examination scores between the control group and the pilot group on the courses leading up to course 5 or course 7, demonstrated the two groups were not significantly different with respect to pervious academic performance (Table 1). The results demonstrated that the students participating in the distributed course 5 pilot project were not statistically different with respect to academic achievement as compared to their counter parts at the main University of Calgary site (Table 2). These results were reproduced in the Course 7 pilot project (Table 2).

**Table 1:** Cumulative percent grades of pilot and control student cohorts based on courses 1–4, for the Course 5 pilot, and courses 1–6, for the Course 7 pilot

	**Pilot group% (SD)**	**Control group% (SD)**	***t***	***p***
**Course 5**	82.2 (5.34)	82.2 (5.22)	0.03	*p* > 0.05
	(n = 11)	(n = 44)		
**Course 7**	81.4 (5.93)	81.1 (5.19)	0.15	*p* > 0.05
	(n = 12)	(n = 48)		

**Table 2:** Academic performance of pilot and control cohorts during each of course 5 and course 7

	**Pilot group% (SD)**	**Control group% (SD)**	***t***	***p***
**Course 5**	84.3 (4.57)	84.3 (5.28)	0.02	*p* > 0.05
	(n = 11)	(n = 44)		
**Course 7**	83.8 (4.57)	82.0 (5.47)	1.20	*p* > 0.05

To investigate potential motivation effects amongst pilot students, mean change scores were calculated between each participant’s cumulative pre course GPA, and the GPA obtained on course 5 or course 7. There was no significant difference between the mean change score of the course 5 pilot participants, *M* = -2.1, *SD* = 4.79, as compared to the control participants, *M* = -2.0, *SD* = 4.21 (*t* = -0.06, *p* > 0.05). The same trend was observed with the course 7 pilot, *M* = -2.5, *SD* = 3.58, and control participants, *M* = -0.9, *SD* = 4.92 (*t* = -1.24, *p* > 0.05).

The feedback provided from the pilot students in both courses was positive towards the program. Two of the themes consistently highlighted were the positive role of the preceptors in the learning experience, and the social connections. This is notable, as the students did not report a sense of isolation despite being separated from their class at the main site.

The students described the preceptors at the rural sites as knowledgeable and enthusiastic both in teaching the course material and providing information about rural practice:

*The preceptors were very enthusiastic and had interesting stories about when things go right, when things go wrong, how you work around the system and, interacting with physicians in the larger centres.* (Course 5 Participant, Site A)

*I appreciated doing clinical correlation with a general practitioner as it felt more reflective to what is expected from me as a student.* (Course 7 Participant, Site A)

The positive social experiences reported by the students centered around the small group learning, and the bonding experience of being removed from the main site as a group. Participants from both courses described the social experience positively.

*…probably the best part. The five of us got along great and we were all friends; we spent a lot of time together outside of class eating lunch or studying or whatever.* (Course 5 Participant, Site B)

*…smaller core groups was nice – [it was] more relaxed and [you were] able to ask more questions* (Course 7 Participant, Site A)*.*

There was initial concern among the program organizers that the students would feel isolated, but feedback from the students suggested this was not the case. The development of relationships with other medical students and the physicians at the rural sites was the primary reason participants reported not feeling isolated:

*Well yes and no. I mean, it was isolating in a sense that we were away from the 180 students that are here at Calgary, but then it was a lot more connected as well because our small group got along really well, so we got to know each other a lot better and we did a lot more things together than we did here in Calgary with everyone else.* (Course 5 Participant, Site B).

Both groups of participants indicated that, in addition to communicating within their small groups, they also used social media websites to mitigate isolation and remain connected with the main site.

## Discussion

The University of Calgary PNME pilot is a unique approach to distributed preclinical education that is distinct from the “satellite campus” approach. This method delivers academic content at a rural community without a dedicated stand-alone medical education facility and integrates a network of rural teaching sites into a pre-existing, single site medical school. The results from the matched cohort trials described here suggest that students are not negatively impacted academically by learning at a distributed site. Neither was there evidence that the pilot group’s academic performance was substantially affected by an underlying motivation factor related to their status as pilot participants. There was no detectable difference in academic performance between the group of students at the main site and the groups at the pilot sites. Additionally, the students provided very positive feedback with respect to the quality of the preceptors, and the social experiences.

These results suggest that an academically equivalent and socially positive learning experience is possible within this form of distributed undergraduate medical education model. By using existing infrastructure and leveraging educational technology, an existing medical education curriculum can be delivered in a wide variety of locations. However, depending on the site selected, the availability of patients and preceptors may be an important limiting factor to implementing this approach.

Our analysis of the distributed learning experience is limited by the sample size of students, courses, and institutions involved. Additionally, these results do not provide any insight as to whether academic equivalence, or positive social learning experiences, would be retained throughout multiple, consecutive, distributed courses. The analysis may also be confounded by selection bias, as participants were chosen from a group who self-elected out of personal interest to be involved in the pilot. It has been demonstrated that medical students with high levels of personal interest in a particular task or topic will perform academically stronger than those who are less personally interested [13]. Therefore, although the groups were equivalent academically, the students who participated in the pilot may be predisposed towards the distributed form of learning and less interested students may not have performed as well at the distributed sites. While these factors prevent us from drawing generalizable conclusions regarding equivalence between the two forms of education delivery, the results are promising and suggest that community based preclinical education programs can be viably delivered in communities previously not considered. These results further suggest that an existing traditional medical education curriculum has the flexibility to be adapted to this form of distributed education.

The benefits of this, rural community based approach extends from the evidence that positive rural learning experiences may increase the number of physicians pursuing rural practices [3,5,7,8,10]. This concept is consistent with social based learning theory in which students’ perceptions of competence and skill are shaped by the site in which they initially develop those skills [14]. These early experiences may be responsible for shaping their later practice location decisions.

The preliminary findings suggest that the networked approach may constitute a viable step towards addressing rural health care disparities without the significant resource investments required for construction of standalone facilities. The future development of the PNME approach will consist of an expanded review of courses for networked delivery. Longitudinal cohort studies evaluating the long term comparative academic performance and practice decision outcomes are the next logical step in investigating the value this model of distributed medical education.

Future investigations are also needed to understand the perspectives and motivations of communities who participate in this initiative. While they may eventually benefit from increased physician retention, the time committed to teaching learners could adversely affect the community’s access to health care. Alternatively, the educational presence may serve to grow the breadth of medical services available to the smaller sites, as well as inspire local high school students towards pursuing medicine. Studies aimed at understanding the benefits and drawbacks of this initiative will be an important step in engaging community participation, and maximizing the benefits of the networked based medical education approach.

## Conclusion

These results suggest that community distributed education in pre-clerkship may offer academically equivalent training to existing traditional medical school curricula while also providing learners with positive rural social learning environments. The approach described may offer the potential to increase exposure to rural practice without the cost of constructing additional physical learning sites.

## Competing interests

The primary author was responsible to report results of program to the funder.

## Authors’ contributions

Dr. DM contributed substantially to the conception, data acquisition, analysis and authoring of this manuscript. He revised and approved the manuscript. PA contributed substantially to the data analysis and writing of the manuscript. Nathan Turley participated in data analysis for this study as well as in the drafting, revising, and approval of the manuscript. Dr. RS, contributed to the project conception, data analysis, and in the revision and approval of the manuscript. Dr. WW participated in the project conception, contributed substantially to the data analysis, and revised and approved the final manuscript. All authors read and approved the final manuscript.

## Pre-publication history

The pre-publication history for this paper can be accessed here:

http://www.biomedcentral.com/1472-6920/14/166/prepub
